# Early Vascular Aging and Subclinical Myocardial Deformation in Children with β-Thalassemia Major: The Role of Asymmetric Dimethylarginine

**DOI:** 10.3390/children13040461

**Published:** 2026-03-27

**Authors:** Pelin Kosger, Zeynep Canan Özdemir, Ayse Sulu, Özcan Bör, Birsen Uçar

**Affiliations:** 1 Department of Pediatric Cardiology, Faculty of Medicine, Eskisehir Osmangazi University, Eskisehir 26000, Turkey; 2 Department of Pediatric Hematology and Oncology, Faculty of Medicine, Eskisehir Osmangazi University, Eskisehir 26000, Turkey; 3 Department of Pediatric Cardiology, Faculty of Medicine, Gaziantep University, Gaziantep 27000, Turkey

**Keywords:** β-thalassemia major, early vascular aging, asymmetric dimethylarginine, myocardial deformation, endothelial dysfunction

## Abstract

Background: Children with β-thalassemia major (β-TM) survive longer due to advances in transfusion and chelation therapy; however, cardiovascular complications have emerged as a leading cause of long-term morbidity. Chronic hemolysis, oxidative stress, and iron overload may promote early endothelial dysfunction and premature vascular aging, yet their impact on myocardial deformation in pediatric patients remains incompletely characterized. Objectives: To evaluate subclinical myocardial dysfunction and arterial stiffness in children with β-TM and to investigate hemolysis-related changes in asymmetric dimethylarginine (ADMA) and L-arginine as biomarkers of endothelial dysfunction in relation to cardiovascular involvement. Methods: Twenty-four children with β-TM and 20 age-matched healthy controls were included. Cardiac structure and myocardial deformation were assessed by conventional echocardiography, tissue Doppler imaging, and speckle-tracking strain analysis. Arterial stiffness was evaluated using oscillometric pulse wave analysis and bilateral carotid intima–media thickness (CIMT). Serum ADMA and L-arginine levels were measured, and hemoglobin, reticulocyte count, and ferritin levels were recorded. Results: Children with β-thalassemia major demonstrated significantly increased arterial stiffness compared with controls, including higher PWV (4.61 ± 0.37 vs. 4.38 ± 0.31), AIx@75 (augmentation index at 75 bpm) (28.5 ± 8.34 vs. 22.8 ± 6.51), left CIMT [0.45 (0.39–0.51) vs. 0.41 (0.38–0.46)], and right CIMT [0.43 (0.39–0.54) vs. 0.40 (0.34–0.46)]. In addition, patients exhibited reduced global longitudinal strain (−19.3 ± 2.91 vs. −21.84 ± 1.91), prolonged isovolumetric relaxation time [53 (37–71) vs. 45 (37–55)], and elevated E/Em (8.44 ± 2.19 vs. 6.92 ± 1.10). ADMA levels were significantly higher in patients (0.54 ± 0.19 vs. 0.39 ± 0.22) and were positively associated with reticulocyte counts and inversely correlated with hemoglobin levels. In addition, both ADMA and ferritin levels were positively correlated with arterial stiffness indices and left ventricular filling pressures. Conclusions: Children with β-thalassemia major exhibit features suggestive of early cardiovascular aging, including impaired myocardial deformation, diastolic involvement, and increased arterial stiffness. The observed association between ADMA levels and markers of hemolysis, vascular stiffness, and myocardial deformation highlights the potential involvement of endothelial dysfunction in premature myocardial–vascular remodeling. These findings suggest that ADMA may serve as a promising biomarker for early cardiovascular risk in pediatric β-thalassemia major; however, further longitudinal and multi-center studies are needed to confirm its clinical utility for risk stratification.

## 1. Introduction

β-thalassemia major (β-TM) is an autosomal recessive hemoglobinopathy characterized by defective or absent synthesis of one or more β-globin chains, resulting in chronic hemolytic anemia. Lifelong regular red blood cell transfusions are required to maintain adequate hemoglobin levels and to prevent anemia-related clinical complications [1]. Children with β-TM are chronically exposed to intravascular hemolysis, transfusion-related iron overload, and persistent oxidative stress. These interrelated pathophysiological processes are key drivers of endothelial dysfunction, promoting early vascular remodeling and premature vascular aging [2]. Reactive oxygen species generated through iron-catalyzed reactions impair endothelial nitric oxide synthase (eNOS) activity, resulting in reduced nitric oxide (NO) bioavailability and endothelial dysfunction [3]. Under physiological conditions, NO is synthesized from L-arginine by eNOS; however, in β-thalassemia major, chronic intravascular hemolysis and oxidative stress disrupt this pathway. In parallel, free hemoglobin released during hemolysis rapidly scavenges circulating NO, further limiting vasodilatory capacity. Moreover, asymmetric dimethylarginine (ADMA), an endogenous competitive inhibitor of eNOS that interferes with L-arginine-dependent NO synthesis, is increased in hemolytic conditions such as β-thalassemia. Elevated ADMA levels further suppress NO production, leading to a marked reduction in NO bioavailability and amplification of endothelial and vascular dysfunction [4,5].

Recent studies have reported increased circulating ADMA levels in patients with β-TM and have linked elevated ADMA concentrations to adverse cardiovascular remodeling and vascular stiffening [6,7,8]. However, in pediatric populations, integrated investigations simultaneously evaluating ADMA, L-arginine, arterial stiffness, and myocardial deformation remain limited. Consequently, the myocardial–vascular interactions underlying early subclinical cardiovascular involvement in children with β-TM are not yet fully characterized.

The present study was designed to comprehensively evaluate biochemical, vascular, and myocardial markers of early cardiovascular aging in children with β-TM. By integrating serum ADMA and L-arginine measurements with arterial stiffness parameters and advanced echocardiographic deformation imaging, we aimed to elucidate the myocardial–vascular coupling mechanisms underlying early subclinical vascular and myocardial remodeling in this population.

## 2. Materials and Methods

### 2.1. Study Design and Population

This single-center, cross-sectional observational study included 24 children with β-thalassemia major (β-TM) followed at the Pediatric Hematology and Pediatric Cardiology Units of Eskisehir Osmangazi University and 20 age- and sex-matched healthy controls. All participants were evaluated between June 2020 and September 2021. The diagnosis of β-TM was confirmed based on hematological and clinical criteria, and all patients were receiving regular transfusion and chelation therapy.

Exclusion criteria for both groups included congenital heart disease, moderate-to-severe valvular disease, systemic hypertension, diabetes mellitus, chronic kidney or liver failure, thyroid disease, significant arrhythmias, and any systemic condition that could affect cardiac or vascular function.

Controls were recruited from healthy children without chronic disease, regular medication use, or abnormal findings on physical examination, electrocardiography, or echocardiography. Controls were matched to patients based on age and sex to improve comparability. Resting heart rate and blood pressure measurements were recorded and showed no significant differences between groups.

### 2.2. Blood Sampling and Biochemical Measurements

After an overnight fast, venous blood samples were obtained in the morning. In the β-TM group, samples were collected immediately before scheduled transfusion to reflect chronic baseline status. Complete blood count, serum ferritin, plasma asymmetric dimethylarginine (ADMA), and L-arginine levels were measured in both groups; glycated hemoglobin and reticulocyte counts were additionally assessed in patients. Samples were centrifuged and stored at −80 °C until analysis.

#### 2.2.1. Measurement of Serum Asymmetric Dimethylarginine (ADMA)

Serum ADMA concentrations were measured using a commercially available Human Asymmetric Dimethylarginine (ADMA) ELISA kit (MyBioSource Inc., San Diego, CA, USA). Absorbance was read on a Chromate 4300 microplate reader (Awareness Technology Inc., Palm City, FL, USA). Results were expressed in µmol/L.

#### 2.2.2. Measurement of Serum L-Arginine (L-Arg)

Serum L-arginine levels were determined using the Human L-Arginine ELISA kit from the same manufacturer (MyBioSource Inc., San Diego, CA, USA). Absorbance measurements were obtained with the Chromate 4300 microplate reader, and results were reported in µg/mL.

All samples were thawed only once prior to analysis to avoid degradation associated with repeated freeze–thaw cycles.

### 2.3. Echocardiography

#### 2.3.1. Two-Dimensional and Doppler Echocardiography

From standard parasternal long- and short-axis and apical views, left ventricular end-diastolic diameter (LVEDd), interventricular septal thickness in diastole (IVSd), left ventricular posterior wall thickness in diastole (LVPWDd), left ventricular mass index (LVMI), mitral annular plane systolic excursion (MAPSE), tricuspid annular plane systolic excursion (TAPSE), and right ventricular end-diastolic diameter (RVEDd) were measured using two-dimensional and M-mode echocardiography.

Left ventricular ejection fraction (LVEF) was calculated using M-mode echocardiography according to the Teichholz formula. Pulsed-wave Doppler was used to obtain transmitral and transtricuspid inflow velocities; early (E) and late (A) diastolic peak velocities were measured and E/A ratios were calculated.

#### 2.3.2. Tissue Doppler Imaging and Myocardial Performance Index

Mitral annular tissue Doppler imaging (TDI) was performed in the apical four-chamber view with the sample volume placed at the lateral mitral annulus. Systolic (Sm), early diastolic (Em), and late diastolic (Am) myocardial velocities, as well as isovolumic relaxation time (IVRT), isovolumic contraction time (IVCT), and ejection time (ET), were recorded. All TDI measurements were averaged over three consecutive beats.

The left ventricular myocardial performance index (MPI, Tei index) was calculated as an index of global systolic and diastolic function using the following formula:

MPI (Tei index) = (IVCT + IVRT)/ET

#### 2.3.3. Two-Dimensional Speckle-Tracking Strain Echocardiography

Two-dimensional speckle-tracking echocardiography was performed using the Philips EPIQ CVx ultrasound system (Philips Medical Systems, Andover, MA, USA) with vendor-specific software (QLAB 10, Philips Medical Systems, Andover, MA, USA).

For global longitudinal strain (GLS), apical four-chamber (A4C), two-chamber (A2C), and three-chamber (A3C) views were obtained. Endocardial borders were manually traced, and the software automatically generated region-of-interest and longitudinal strain curves. Segmental longitudinal strain values were calculated according to a 17-segment left ventricular model, and GLS was derived as the average of all segmental values.

Global circumferential strain (GCS) was assessed from parasternal short-axis images at basal, mid-ventricular, and apical levels. Circumferential strain curves were generated for each segment, and mean values for each level and for the entire left ventricle were obtained. Strain measurements were averaged from three consecutive cardiac cycles. Segments with inadequate tracking despite manual adjustment were excluded from analysis. The observer performing strain analysis was blinded to the clinical status (patient vs. control) of the participants.

### 2.4. Carotid Intima–Media Thickness (CIMT)

Right and left carotid intima–media thickness (CIMT) was measured ultrasonographically by the same pediatric cardiologist using a high-frequency linear transducer (12 MHz) (Philips Medical Systems, Andover, MA, USA). For each carotid artery, measurements were obtained from the far wall of the common carotid artery, approximately 1 cm proximal to the bifurcation, at end-diastole synchronized with the R wave on the ECG. Three consecutive measurements were recorded for each side, and the mean value was accepted as the CIMT of that artery. The average of right and left CIMT values was used as the overall CIMT for statistical analysis. Top of Form

### 2.5. Pulse Wave Analysis Bottom of Form

Pulse wave analysis was performed using the Mobil-O-Graph device (Industrielle Entwicklung Medizintechnik und Vertriebsgesellschaft mbH, Stolberg, Germany). An appropriately sized cuff was placed on the non-dominant arm of each participant, and the device was programmed to perform a 24 h ambulatory assessment according to the manufacturer’s recommendations. During the monitoring period, the device automatically recorded measurements at predefined intervals throughout daytime and nighttime. Artefactual or technically inadequate readings were automatically identified and excluded from the analysis. At the end of the 24 h monitoring period, the device software generated averaged 24 h values for peripheral systolic and diastolic blood pressure, central systolic and diastolic blood pressure, cardiac index, augmentation index (AIx@75), pulse wave velocity (PWV), and cardiac output (CO). These averaged parameters were used for statistical analysis.

### 2.6. Statistical Analysis

The distribution of continuous variables was assessed using the Shapiro–Wilk test. Normally distributed data are presented as mean ± standard deviation, whereas non-normally distributed data are presented as median (interquartile range). Categorical variables are expressed as frequencies and percentages. Between-group comparisons were performed using the independent samples t-test or the Mann–Whitney U test, as appropriate. Categorical variables were compared using the chi-square test. Correlations were evaluated using Pearson or Spearman correlation coefficients, according to data distribution. All statistical analyses were conducted using SPSS version 21.0 (IBM Corp., Chicago, IL, USA), and a two-sided *p* value < 0.05 was considered statistically significant.

Post hoc power analyses were performed for ADMA and GLS based on observed effect sizes (Cohen’s d) using a two-sided α of 0.05. The achieved power was approximately 0.70 for ADMA (Cohen’s d = 0.73) and 0.92 for GLS (Cohen’s d = 1.01).

Given the exploratory nature of the study and the large number of echocardiographic and biochemical variables analyzed, no formal adjustments for multiple comparisons were applied. Therefore, the results should be interpreted as hypothesis-generating.

## 3. Results

### 3.1. Clinical and Biochemical Characteristics

The mean age of children with β-thalassemia major was 13.6 ± 5.1 years; 9 were girls and 15 were boys. There were no significant differences between the patient and control groups regarding age, sex, body weight, height, body mass index, resting heart rate, or blood pressure (all *p* > 0.05).

Hemoglobin levels were significantly lower in the β-thalassemia major group than in controls (*p* < 0.001). The median ferritin concentration in patients was 1217 ng/mL (interquartile range: 903–2829), with values ranging from 181 to 6722 ng/mL. Plasma ADMA levels were significantly higher in patients compared with controls (*p* = 0.02), whereas L-arginine levels did not differ between groups (Table 1).

### 3.2. Echocardiographic Findings

On conventional echocardiography, left ventricular mass index (LVMI), mitral E and A wave velocities, the E/Em ratio, and IVRT measured by tissue Doppler imaging were all significantly higher in the β-thalassemia major group compared with controls (*p* < 0.001, *p* = 0.037, *p* = 0.05, *p* = 0.008, and *p* = 0.007, respectively).

Speckle-tracking analysis showed that left ventricular global longitudinal strain (GLS) was markedly reduced in patients compared with controls (*p* = 0.007) (Table 2).

### 3.3. Vascular Function and Pulse Wave Analysis

Pulse wave analysis revealed that AIx@75, pulse wave velocity (PWV), pulse pressure, cardiac output (CO), and both right and left carotid intima–media thickness (CIMT) values were significantly higher in the β-thalassemia major group than in controls (*p* = 0.02, *p* = 0.05, *p* = 0.002, *p* = 0.001, *p* = 0.017, and *p* = 0.02, respectively). In contrast, peripheral and central diastolic blood pressures were lower in patients (*p* = 0.015 and *p* = 0.05) (Table 3).

### 3.4. Correlation Analyses

Serum ADMA levels demonstrated significant positive correlations with left ventricular mass index (LVMI) (r = 0.371, *p* = 0.031), the E/Em ratio (r = 0.795, *p* < 0.001), right carotid intima–media thickness (CIMT) (r = 0.346, *p* = 0.021), left CIMT (r = 0.441, *p* = 0.005), pulse pressure (r = 0.317, *p* = 0.044), pulse wave velocity (PWV) (r = 0.367, *p* = 0.020), and reticulocyte count (r = 0.795, *p* < 0.001) (Table 4). In contrast, ADMA levels were inversely correlated with hemoglobin levels (r = −0.344, *p* = 0.026). Ferritin levels were positively correlated with the E/Em ratio (r = 0.588, *p* < 0.001). Additionally, LVMI showed a positive correlation with isovolumetric relaxation time (IVRT) (r = 0.378, *p* = 0.010), and global longitudinal strain (GLS) was positively correlated with hemoglobin levels (r = 0.502, *p* = 0.005).

## 4. Discussion

In this cohort of children with thalassemia, we identified a distinct pattern of early and multisystemic cardiovascular involvement when compared with healthy peers. Diastolic function was impaired, as reflected by prolonged IVRT, altered E and A velocities, and an increased E/Em ratio, while reduced global longitudinal strain indicated subtle myocardial deformation abnormalities despite preserved conventional systolic indices. In parallel, oscillometric pulse wave analysis revealed lower peripheral and central diastolic blood pressure in the presence of higher pulse wave velocity, augmentation index, and cardiac output, consistent with increased arterial stiffness. Furthermore, bilateral increases in mean carotid intima–media thickness pointed to incipient vascular remodeling. The observed correlations of these myocardial and vascular changes with ADMA and ferritin levels suggest a potential involvement of endothelial dysfunction and iron overload in cardiovascular alterations in pediatric thalassemia.

In hemolytic disorders, circulating ADMA concentrations may be substantially influenced by erythrocyte turnover, as red blood cells represent an important storage compartment for ADMA. Increased erythrocyte destruction therefore results in excess free ADMA release into the circulation [9]. Accordingly, the positive correlation between ADMA and reticulocyte counts and the negative correlation with hemoglobin levels suggests that hemolysis may contribute to ADMA elevation. In line with our findings, Mohammad et al. reported significantly increased ADMA levels in children with β-thalassemia major compared with healthy controls and demonstrated a significant association with pulmonary artery pressure, consistent with a potential role of ADMA-mediated endothelial dysfunction in the hemodynamic alterations observed in this disease [7].

Clinical studies have consistently demonstrated increased arterial stiffness, elevated pulse wave velocity, and increased carotid intima–media thickness in children and young adults with β-thalassemia major, even in the absence of overt cardiovascular disease, and these vascular abnormalities have been closely linked to ADMA levels, consistent with a potential role of endothelial dysfunction in premature vascular aging [6,10]. In our cohort, both PWV and CIMT were significantly higher in children with β-thalassemia major than in healthy controls, indicating reduced arterial compliance and early structural vascular remodeling. Higher ADMA levels were significantly associated with increased PWV, augmentation index, and cardiac output, while both central and peripheral diastolic blood pressures were lower, consistent with impaired arterial elastic recoil and earlier return of reflected waves. The concomitant reduction in diastolic blood pressure further supports impaired arterial elastic recoil and may contribute to subclinical myocardial hypoperfusion and deformation, whereas the increase in cardiac output likely reflects a compensatory response to chronic anemia and reduced oxygen delivery. In patients with β-thalassemia major, chronic iron overload secondary to frequent blood transfusions represents one of the principal mechanisms underlying myocardial structural and functional impairment. Serum ferritin levels above 2500 ng/mL are considered to indicate high cardiac risk, whereas levels below 1000 ng/mL reflect adequate iron control [11]. In our cohort, the strong positive correlation between ferritin levels and the E/Em ratio suggests that iron overload may serve as a biochemical marker of early diastolic dysfunction even in the presence of preserved ejection fraction. Importantly, E/Em was also positively correlated with ADMA levels, and circulating ADMA concentrations were significantly higher in patients than in controls, consistent with a potential contribution of endothelial dysfunction to increased left ventricular filling pressures in β-thalassemia. The close association between ADMA levels and reticulocyte counts further highlights the contribution of ongoing hemolytic activity to nitric oxide pathway disruption and endothelial injury. Hemolysis-driven nitric oxide depletion and ADMA-mediated endothelial nitric oxide synthase inhibition may act together to promote microvascular dysfunction and impair myocardial perfusion, potentially compromising active ventricular relaxation. Although relative wall thickness remained within the normal range, the observed increase in left ventricular mass index (LVMI), together with its significant positive correlations with both E/Em ratio and isovolumetric relaxation time (IVRT), is consistent with a pattern of eccentric left ventricular remodeling associated with early impairment of diastolic relaxation. These findings suggest that subclinical myocardial hypertrophy in our patients is accompanied by early abnormalities in ventricular relaxation mechanics. Notably, the significant association between ADMA levels and LVMI is consistent with a potential role of endothelial dysfunction in structural myocardial remodeling, indicating that ADMA may contribute not only to functional diastolic impairment but also to the development of myocardial hypertrophy in this population. A significant association between ADMA levels and left ventricular hypertrophy has also been demonstrated across various patient populations, supporting a broader pathophysiological role of ADMA in myocardial structural remodeling [12,13].

Our findings suggest that left ventricular global longitudinal strain (GLS) was significantly reduced in asymptomatic children with β-thalassemia major compared with healthy peers, potentially indicating early subclinical systolic impairment despite preserved ejection fraction. This result is consistent with previous studies reporting reduced strain values in young patients with thalassemia, including the cohorts evaluated by Cheung et al. and Piccione et al., both of whom identified impaired myocardial deformation in asymptomatic individuals [14,15]. Similarly, El Razaky et al. reported significantly decreased longitudinal, circumferential, and radial strain values in 100 children with β-thalassemia major, with negative correlations between strain parameters and ejection fraction [16]. In the present study, the observed correlation between GLS and hemoglobin levels may reflect the influence of chronic anemia-related high-output physiology and myocardial hypoxia, which may represent the primary drivers of early impairment in longitudinal myocardial deformation before overt systolic dysfunction becomes evident in this population. Similar associations between hemoglobin levels and global longitudinal strain (GLS) have been reported in previous studies, supporting the concept that anemia severity is closely linked to early impairment of myocardial deformation in chronically anemic patient populations [17,18].

This study has certain limitations. The relatively small sample size and single-center design may limit the generalizability of our findings. The cross-sectional design precludes conclusions regarding causality. In addition, myocardial iron load was not directly assessed using cardiac magnetic resonance T2 imaging; therefore, future longitudinal studies integrating CMR-based iron quantification are warranted to further elucidate the temporal relationship between iron overload, endothelial dysfunction, and myocardial deformation.

## 5. Conclusions

The present study demonstrate that pediatric patients with β-thalassemia major may exhibit a distinct early ventriculo–arterial dysfunction characterized by endothelial impairment, increased arterial stiffness, high-output physiology, reduced global longitudinal strain, and subclinical left ventricular diastolic dysfunction. The strong associations between ADMA, ferritin, arterial stiffness parameters, and echocardiographic markers of diastolic function are consistent with a multifactorial pathophysiological process driven by hemolysis, iron toxicity, and vascular dysfunction. Importantly, the concomitant presence of reduced GLS with impaired relaxation, elevated filling pressures, and increased LV mass may reflect that both systolic deformation and diastolic properties are already compromised despite preserved ejection fraction. These findings support the concept of the need for comprehensive cardiovascular surveillance integrating biochemical, vascular, and advanced echocardiographic markers to enable early identification of high-risk patients and timely initiation of cardioprotective strategies.

## Figures and Tables

**Table 1:** Comparison of anthropometric and laboratory characteristics between children with β-thalassemia major and healthy controls.

Variables	Patients (*n* = 24)	Controls (*n* = 20)	*p*
Age (year)	13.62 ± 5.1	13.55 ± 4.18	0.958
Sex (f/m)	9/15	11/9	0.196
Weight (kg)	40.26 ± 18.16	45.05 ± 16.69	0.279
Height (cm)	141.13 ± 23.01	148.05 ± 17.49	0.376
BMI (kg/m^2^)	19.2 ± 0.36	19.8 ± 0.37	0.614
Resting HR (bpm)	83.75 ± 10.09	84.1 ± 14.42	0.935
Resting SBP (mmHg)	113.6 ± 6.65	103 ± 5.5	0.103
Hb (gr/dL)	8.91 ± 2.23	13.44 ± 1.22	<0.001
Creatinine (mg/dL)	0.5 ± 0.14	0.54 ± 0.15	0.347
AST (u/L)	24.01 ± 7.81	23.89 ± 13.27	0.955
ALT (u/L)	18.16 ± 11.64	13.01 ± 6.47	0.216
ADMA (µmol/L)	0.54 ± 0.19	0.39 ± 0.22	0.02
L-arginin (µg/mL), median (IQR)	259.6 (215.5–265.7)	260.2 (80.7–262.2)	0.953

Data are presented as mean ± standard deviation (SD) or median (interquartile range, IQR) as appropriate. BMI, body mass index; Hb, hemoglobin; HR, heart rate; SBP, systolic blood pressure; AST, aspartate aminotransferase; ALT, alanine aminotransferase; ADMA, asymmetric dimethylarginine. Ferritin levels were measured only in patients and are not included in the comparative analysis. Median ferritin level: 1217 ng/mL.

**Table 2:** Comparison of echocardiographic parameters between children with β-thalassemia major and healthy controls.

Variables	Patients (*n* = 24)	Controls (*n* = 20)	*p*
IVSd (mm)	7.45 ± 1.21	6.92 ± 1.08	0.139
LVEDd (mm)	45.81 ± 6.84	43.72 ± 8.79	0.383
RWT (mm)	0.31 ± 0.08	0.32 ± 0.04	0.855
LVMI (g/m^2^)	90.95 ± 26.13	66.39 ± 13.91	<0.001
EF (%)	69.32 ± 6.7	70.33 ± 5.6	0.621
FS (%)	39.3 ± 4.96	39.5 ± 4.68	0.854
E (cm/s)	106.43 ± 20.6	83.59 ± 15.53	<0.001
A (cm/s)	57.95 ± 12.82	49.83 ± 10.68	0.037
Em (cm/s)	12.4 (8–23.6)	11.75 (8.8–16.2)	0.436
Am (cm/s)	7.31 ± 2.2	6.2 ± 1.37	0.06
Sm (cm/s)	8.22 ± 1.73	7.59 ± 1.45	0.210
E/Em	8.44 ± 2.19	6.92 ± 1.1	0.008
IVRTm, median (IQR)	53 (37–71)	45 (37–55)	0.008
IVCTm	51 ± 9.05	49.85 ± 8.61	0.670
ETm	274 ± 26.9	271.7 ± 32.32	0.724
MPIm	0.38 ± 0.06	0.35 ± 0.06	0.149
MAPSE (mm)	13.58 ± 2.35	13.35 ± 1.16	0.690
TAPSE (mm)	21.28 ± 3.17	20.65 ± 2.52	0.478
RVEDd (mm)	33.13 ± 6.34	31.4 ± 4.2	0.31
TR velocity (m/s)	1.91 ± 0.34	1.78 ± 0.32	0.245
MPIt	0.40 ± 0.09	0.36 ± 0.07	0.204
GLS (%)	−19.3 ± 2.91	−21.84 ± 1.91	0.007
GCS (%)	−21.78 ± 5.49	−22.76 ± 2.89	0.549

Data are presented as mean ± standard deviation (SD) or median (interquartile range, IQR), as appropriate. IVSd: interventricular septal thickness in diastole; LVEDd: left ventricular end-diastolic diameter; RWT: relative wall thickness; LVMI: left ventricular mass index; EF: left ventricular ejection fraction; FS: fractional shortening; E, A: transmitral early and late diastolic velocities; Em, Am, Sm: mitral annular early, late diastolic and systolic velocities; IVRTm: isovolumic relaxation time; IVCTm: mitral isovolumic contraction time; ETm: mitral ejection time; MPI: myocardial performance index; MAPSE: mitral annular plane systolic excursion; TAPSE: tricuspid annular plane systolic excursion; RVEDd: right ventricular end-diastolic diameter; TR velocity: tricuspid regurgitation velocity; GLS: global longitudinal strain; GCS: global circumferential strain.

**Table 3:** Comparison of pulse wave analysis and carotid intima–media thickness parameters between patients with β-thalassemia major and healthy controls.

Variables	Patients (*n* = 24)	Controls (*n* = 20)	*p*
Peripheral SBP (mmHg)	112.04 ± 10.73	109.1 ± 6.34	0.294
Peripheral DBP (mmHg)	62.95 ± 6.44	67.6 ± 5.17	0.05
Peripheral PP (mmHg)	48.71 ± 7.57	41.45 ± 6.09	0.002
Central SBP (mmHg), median (IQR)	98 (84–120)	99 (80–109)	0.347
Central DBP (mmHg)	65.47 ± 6.01	69 ± 5.38	0.05
PWV (m/s)	4.61 ± 0.37	4.38 ± 0.31	0.05
Alx@75	28.5 ± 8.34	22.8 ± 6.51	0.021
CO (L/min)	4.45 ± 0.48	3.8 ± 0.89	0.02
Left CIMT (mm), median (IQR)	0.45 (0.39–0.51)	0.41 (0.38–0.46)	0.001
Right CIMT (mm), median (IQR)	0.43 (0.39–0.54)	0.40 (0.34–0.46)	0.017

SBP: systolic blood pressure; DBP: diastolic blood pressure; PWV: pulse wave velocity; AIx@75: augmentation index normalized to a heart rate of 75 beats/min; CO: cardiac output; CIMT: carotid intima–media thickness. Data are presented as mean ± standard deviation (SD) or median (interquartile range, IQR), as appropriate.

**Table 4:** Correlations between serum ADMA levels and cardiovascular parameters.

Variables	Correlations	*p*
LVMI	r: 0.371	0.031
E/Em	r: 0.795	0.001
Right CIMT	rs: 0.346	0.021
Left CIMT	rs: 0.441	0.005
PWV	r: 0.367	0.02
Reticulocyte	rs: 0.795	0.001

ADMA: asymmetric dimethylarginine; LVMI: left ventricular mass index; CIMT: carotid intima–media thickness; PWV: pulse wave velocity; r: Pearson correlation coefficient; rs: Spearman correlation coefficient.

## Data Availability

The data presented in this study are available on request from the corresponding author.

## References

[1] Kwiatkowski J.L., Lanzkowsky P., Lipton J.M., Fish J.D. (2016). Hemoglobinopathies. Lanzkowsky’s Manual of Pediatric Hematology and Oncology.

[2] Trudel E., Felfly M., Lemsaddek W., Garcia D., Cloutier G. (2012). Vascular endothelial dysfunction in β-thalassemia occurs despite increased eNOS expression and preserved vascular smooth muscle cell reactivity to nitric oxide. PLoS ONE.

[3] Frimat M., Boudhabhay I., Roumenina L.T. (2019). Hemolysis-derived products toxicity and endothelium: Model of the second hit. Toxins.

[4] Moncada S., Higgs A. (1993). The L-arginine–nitric oxide pathway. N. Engl. J. Med..

[5] Hahalis G., Kremastinos D.T., Terzis G., Kalogeropoulos A.P., Chrysanthopoulou A., Karakantza M., Kourakli A., Adamopoulos S., Tselepis A.D., Grapsas N. (2008). Global vasomotor dysfunction and accelerated vascular aging in β-thalassemia major. Atherosclerosis.

[6] Gursel O., Tapan S., Sertoglu E., Taşçılar E., Eker I., Ileri T., Uysal Z., Kurekci A.E. (2018). Elevated plasma asymmetric dimethylarginine levels in children with β-thalassemia major may be an early marker for endothelial dysfunction. Hematology.

[7] El-Shanshory M., Ibrahim B., Amr D., El-Kholy N., Mokhtar M. (2014). Asymmetric dimethylarginine levels in children with β-thalassemia and their correlations with tricuspid regurgitant jet velocity. Pediatr. Blood Cancer.

[8] Omar I.M.A., Safwat N.A., Kenny M.A., Abdel-Maksoud M.M.M. (2023). Assessment of asymmetric dimethylarginine level in pediatric patients with β-thalassemia: Relation to cardiovascular complications. QJM.

[9] Benites B.D., Olalla-Saad S.T. (2019). An update on arginine in sickle cell disease. Expert Rev. Hematol..

[10] Cheung Y.F., Chow P.C., Chan G.C., Ha S.Y. (2006). Carotid intima–media thickness is increased and related to arterial stiffening in patients with β-thalassemia major. Br. J. Haematol..

[11] Taher A.T., Musallam K.M., Cappellini M.D. (2021). β-Thalassemias. N. Engl. J. Med..

[12] Zoccali C., Mallamaci F., Maas R., Benedetto F.A., Tripepi G., Malatino L.S., Cataliotti A., Bellanuova I., Böger R., on behalf of The Creed Investigators (2002). Left ventricular hypertrophy, cardiac remodeling and asymmetric dimethylarginine in hemodialysis patients. Kidney Int..

[13] Sibal L., Agarwal S.C., Home P.D., Boger R.H. (2010). The role of asymmetric dimethylarginine in endothelial dysfunction and cardiovascular disease. Curr. Cardiol. Rev..

[14] Cheung Y.F., Liang X.C., Chan G.C., Wong S.J., Ha S.Y. (2010). Myocardial deformation in patients with β-thalassemia major: A speckle tracking echocardiographic study. Echocardiography.

[15] Cusmà-Piccione M., Piraino B., Zito C., Khandheria B.K., Di Bella G., De Gregorio C., Oreto L., Rigoli L., Ferraù V., Salpietro C.D. (2013). Early identification of cardiovascular involvement in patients with β-thalassemia major. Am. J. Cardiol..

[16] El Razaky O.A., El-Shanshory M.R., El-Shehaby W.A., Hables N.M., Elshamia A.M., Fayed A.M., El-Kholy A.M., El-Dosoky E.A. (2019). Left ventricular regional function in children with β-thalassemia with no cardiac manifestations: A four-dimensional echocardiographic study. Indian J. Hematol. Blood Transfus..

[17] Hu B.Y., Yang Z.G., Tang S.S., Wen X.L., Yan W.F., Yu S.Q., Li Y. (2025). Impact of anemia on left ventricular function and deformation in patients with essential hypertension: A cardiac magnetic resonance study. Quant. Imaging Med. Surg..

[18] Qian W.L., Xu R., Shi R., Li Y., Guo Y.K., Fang H., Jiang L., Yang Z.G. (2023). Worsening effect of anemia on left ventricular function and global strain in type 2 diabetes mellitus patients: A 3.0-T CMR feature-tracking study. Cardiovasc. Diabetol..

